# Non-motor tasks improve adaptive brain-computer interface performance in users with severe motor impairment

**DOI:** 10.3389/fnins.2014.00320

**Published:** 2014-10-14

**Authors:** Josef Faller, Reinhold Scherer, Elisabeth V. C. Friedrich, Ursula Costa, Eloy Opisso, Josep Medina, Gernot R. Müller-Putz

**Affiliations:** ^1^Laboratory of Brain-Computer Interfaces, Institute for Knowledge Discovery, Graz University of TechnologyGraz, Austria; ^2^Cognitive Neuroscience Lab, University of California, San DiegoSan Diego, CA, USA; ^3^Department of Functional Rehabilitation, Guttmann Institute, Neurorehabilitation University Institute Affiliated with the UABBarcelona, Spain; ^4^Health Science Research Institute, “Germans Trias i Pujol” FoundationBarcelona, Spain

**Keywords:** adaptive brain-computer interface (BCI), stroke, spinal cord injury (SCI), event-related desynchronization (ERD), electroencephalography (EEG), assistive technology, mental tasks

## Abstract

Individuals with severe motor impairment can use event-related desynchronization (ERD) based BCIs as assistive technology. Auto-calibrating and adaptive ERD-based BCIs that users control with motor imagery tasks (“*SMR-AdBCI*”) have proven effective for healthy users. We aim to find an improved configuration of such an adaptive ERD-based BCI for individuals with severe motor impairment as a result of spinal cord injury (SCI) or stroke. We hypothesized that an adaptive ERD-based BCI, that automatically selects a user specific class-combination from motor-related and non motor-related mental tasks during initial auto-calibration (*“Auto-AdBCI”*) could allow for higher control performance than a conventional *SMR-AdBCI*. To answer this question we performed offline analyses on two sessions (21 data sets total) of cue-guided, five-class electroencephalography (EEG) data recorded from individuals with SCI or stroke. On data from the twelve individuals in Session 1, we first identified three bipolar derivations for the *SMR-AdBCI*. In a similar way, we determined three bipolar derivations and four mental tasks for the *Auto-AdBCI*. We then simulated both, the *SMR-AdBCI* and the *Auto-AdBCI* configuration on the unseen data from the nine participants in Session 2 and compared the results. On the unseen data of Session 2 from individuals with SCI or stroke, we found that automatically selecting a user specific class-combination from motor-related and non motor-related mental tasks during initial auto-calibration (*Auto-AdBCI*) significantly (*p* < 0.01) improved classification performance compared to an adaptive ERD-based BCI that only used motor imagery tasks (*SMR-AdBCI*; average accuracy of 75.7 vs. 66.3%).

## 1. Introduction

Electroencephalography (EEG) based brain-computer interfaces (BCIs) can restore communication for severely impaired individuals (Birbaumer et al., 1999; Millán et al., 2010). Here, we focus on BCIs that operate based on the dynamics of oscillatory bioelectrical brain activity. These BCIs exploit the fact that performing motor imagery or other specific mental tasks leads to spatio-spectrally specific power decreases (event-related desynchronization, ERD) or increases (event-related synchronization, ERS) in the EEG (Pfurtscheller and Lopes da Silva, 1999). ERD-based BCIs use signal processing and statistical machine learning techniques to translate patterns of such power changes into control signals.

Operating ERD-based BCIs is a skillful action and requires initial system calibration and user training of varying extent (Allison and Neuper, 2010). Conventional calibration and training paradigms require (a) recording EEG while users perform cue-guided mental activity, (b) offline training of a pattern recognition system, followed by (c) feedback training based on the computed classifier. Typically, the feedback training data is (d) reanalyzed offline to create a more accurate and robust classifier. The common practice of reiterating steps (c) and (d) over multiple training sessions has been shown to lead to effective control even for users with motor impairment (Pfurtscheller et al., 2000; Neuper et al., 2003; Wolpaw and McFarland, 2004; Kübler et al., 2005; Müller-Putz et al., 2005). This approach, however, can be time-consuming and strenuous, especially for users with severe motor impairment.

Using a high number of electrodes with this conventional training approach, has been shown to allow for high control proficiency for healthy users after only one day of training (e.g., Blankertz et al., 2008). Increased setup time, higher user discomfort and higher cost, however, render this approach slightly less practical for clinical and home applications.

In contrast to conventional training approaches, adaptive ERD-based BCI training paradigms provide feedback based on the user's brain activity as early as possible and allow both the user and the system to continuously adapt to each other. In healthy users, adaptive ERD-based BCI training paradigms have been shown to work effectively with both, a low (Vidaurre et al., 2006; Faller et al., 2012b) and a high (Vidaurre et al., 2011) number of EEG electrodes.

Another way to improve the performance of ERD-based BCIs is to optimize the user's control strategy: Selecting a user specific combination of mental tasks for example has been shown to boost control proficiency (Obermaier et al., 2003; Blankertz et al., 2008; Galán et al., 2008). In a similar way, combining motor related control tasks with non-motor related tasks proved as another effective strategy to improve performance Friedrich et al., 2012, 2013; Scherer et al., 2013 in healthy individuals.

We aim to identify a general configuration (three bipolar channels and four mental tasks) for an easy-to-use, auto-calibrating and adaptive ERD-based BCI that auto-selects a user-specific task combination and allows for robust control after a short training time for a large percentage of users with severe motor impairment as a result of spinal cord injury (SCI) or stroke. We used three bipolar derivations for our system because this configuration has proven effective in a large number of studies both for healthy users (e.g., Scherer et al., 2008 or an Adaptive BCI in Vidaurre et al., 2006) and users with motor impairment (e.g., Müller-Putz et al., 2005; Mohapp et al., 2006). Our design gives preference to bipolar (Vidaurre et al., 2006) over Laplacian (Faller et al., 2012b) derivations to require fewer electrodes and hence make the system more practical for clinical and sustained home use by individuals with severe motor impairment. Generally, screening users with more classes increases the chance of effective BCI control. We decided to limit the number of mental tasks to four because of reasons of practicality and usability. With four classes, our system would typically auto-calibrate in less than 6 min.

Inferring from the knowledge with healthy users outlined above, we hypothesized that auto-selecting a user specific class combination of motor-related and non motor-related mental tasks during initial auto-calibration of an adaptive ERD-based BCI (“*Auto-AdBCI*”) could increase performance in comparison to an adaptive ERD-based BCI that uses only standard motor imagery tasks (“*SMR-AdBCI*”) in individuals with SCI or stroke.

To answer this question, we performed offline analyses on two sessions of 30 channel EEG data from 13 individuals with severe motor impairment as a result of SCI or stroke. On the data from Session 1, we identified the general configuration for the *Auto-AdBCI* by running a minimal adaptive BCI configuration (“*Mini-AdBCI*”) for all combinations of every single bipolar derivation and every single class combination and selecting the three channels and four classes that yielded the highest performance. In the same way, we also identified three bipolar derivations for the standard *SMR-AdBCI*. On the data from Session 2, we then simulated both, the *Auto-AdBCI* and the standard *SMR-AdBCI* configuration and compared the performance results.

## 2. Materials and methods

### 2.1. EEG signal acquisition

**Figure 1:**
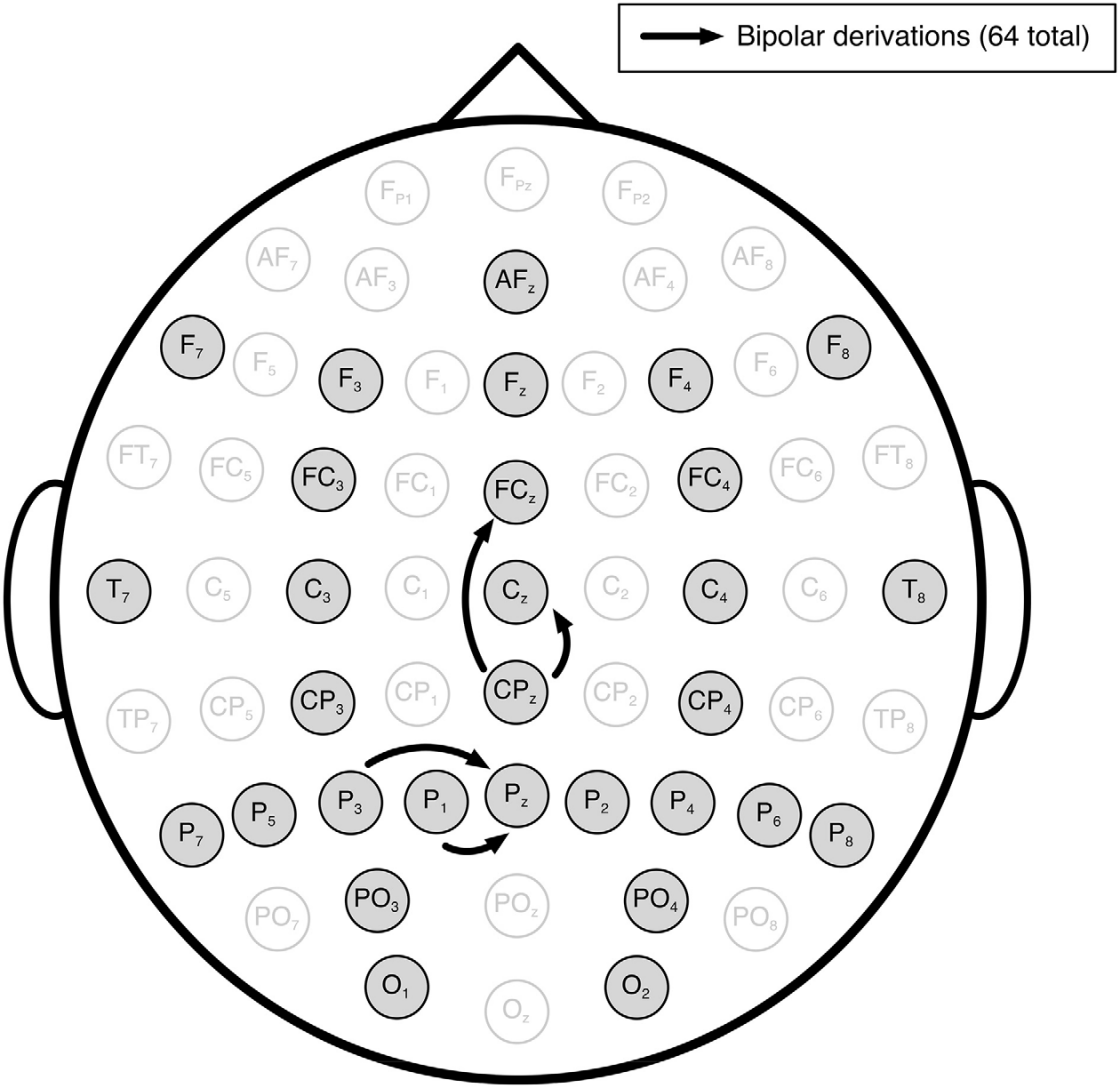
**Locations of the 30 EEG electrodes recorded in our study**. In total, 64 bipolar derivations were used in our analyses. The bipolars were in sagittal and coronal orientation with one or no electrode positions as gaps in between (four representative examples indicated by the black arrows).

We recorded EEG from the 30 scalp locations illustrated in Figure 1 (International 10/20 System of Electrode Placement). The reference and ground electrodes were attached to the left ear-lobe and right mastoid respectively. All signals were recorded using active electrodes and a biosignal amplifier (g.USBamp, Guger Technologies OG, Graz, Austria). The signal was sampled at 256 Hz, with a band-pass filter between 0.5 and 100 Hz and a notch filter at 50 Hz.

### 2.2. Participants

We recorded two sessions of EEG data from 13 volunteers with severe motor impairment (age 39.1 ± 9.1; 7 female) at the Institut Guttmann Neurorehabilitation Hospital (Barcelona, Spain). Seven of the volunteers were diagnosed with SCI (injury between C3 and C5, ASIA A to C, according to Maynard et al., 1997) and six with different types of stroke. The participants S05 and S09 were in “locked-in state (LIS)” according to the definition in Kübler and Birbaumer (2008). Two participants, S04 and S13 were left-handed, the others right-handed. Four participants could not participate in Session 2. Two of them became ill (respiratory infection; severe pressure sore) and the other two did not have time to come in for the second measurement within the 2 week recording period because of other appointments. We had to exclude the data of participant S13, because it was strongly congested with artifacts. This left data of twelve participants in Session 1 and nine participants in Session 2 for analysis. See Table 1 for more details. The study, including measurement protocol and consent procedure, were approved by the local ethics board, “Comitè d'Ètica Assistencial de l'Institut Guttmann.” Written, informed consent was obtained for every participant. In many cases, written consent had to be provided by the participants' legal representatives as many of the participants were not able to write due to motor impairment. The participants were instructed about the paradigm in person by caregivers with the support of presentation slides and other written briefing material.

**Table 1:** **Detailed information about the 13 participants with severe motor impairment**.

**User**	**Sex**	**Age (years)**	**Months since injury**	**Pathology**	**Functional disability**
S01	F	43	27	SCI at C5, ASIA C	Tetraplegia
S02	M	38	15	SCI at C4, ASIA A	Tetraplegia
S03	M	36	53	SCI at C5, ASIA A	Tetraplegia
S04	F	33	2	SCI at C5, ASIA C	Tetraplegia
S05	M	42	6	Brainstem stroke	Locked-in state
S06^‡^	M	45	26	Brainstem stroke	Tetraplegia
S07	F	31	5	Brainstem stroke	Locked-in state
S08^‡^	F	40	255	SCI at C5, ASIA A	Tetraplegia
S09	F	57	5	Hemorrhagic stroke, left hemisphere	Global aphasia; right hemiparesis
S10	M	37	13	SCI at C3, ASIA A	Tetraplegia
S11^‡^	M	50	15	SCI at C4, ASIA A	Tetraplegia
S12	F	20	6	Bilateral, intracerebral hemorrhagic stroke	Tetraparesis
S13^‡^	F	36	58	Basal ganglia and brainstem stroke	Tetraparesis
Mean		39.1	37.4		
*SD*		9.1	67.8		

The symbol

‡ indicates, which volunteers were not able to participate in the second session. The data of participant S13 was excluded, because it was too strongly congested with artifacts.

### 2.3. Experimental paradigm

**Figure 2:**
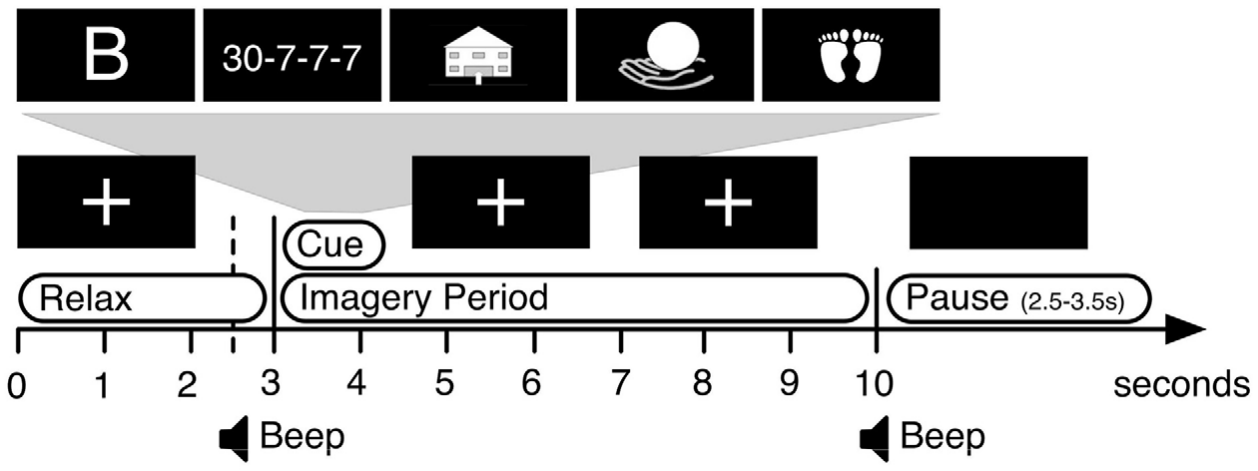
**Schematic depiction of the structure of one single trial**.

We used a modified cue-guided Graz-BCI paradigm (Pfurtscheller and Neuper, 2001, see Figure 2). The participants were instructed to perform one of five different specific mental tasks starting from the appearance of the visual cue until the disappearance of the cue and the cross seven seconds later. Two of the mental tasks were motor-related: Sustained imagery of (1) a dorsiflexion of both feet (“*Feet*”) and (2) a palmar grasp of the right hand (“*Hand*”). The other three classes were non motor-related tasks: For condition (3), participants were instructed to mentally recall as many words as possible starting with a provided letter (“*Word*”). The letters were drawn from a uniform random distribution over the custom alphabet A, D, E, F, G, H, I, J, C, M, N, O, P, R, S, T, L, and V (adapted for Spanish language). For condition (4), participants were instructed to subtract a given subtrahend (randomly between 3 and 10) from a given minuend (randomly between 15 and 30) and to keep subtracting the subtrahend from the last difference (e.g., 17 − 9 = 8 ⇒ 8 − 9 = −1 ⇒ −1 − 9 = −10, etc.) for the duration of the imagery period (“*Math*”). For condition (5), participants were instructed to mentally navigate through a well known building (“*Nav*”). During each run (6 min long), we recorded 25 trials, five for each of the five cue conditions. The sequence of cues was random. In every session we recorded eight runs (i.e., 200 trials per session).

### 2.4. Analyses

**Figure 3:**
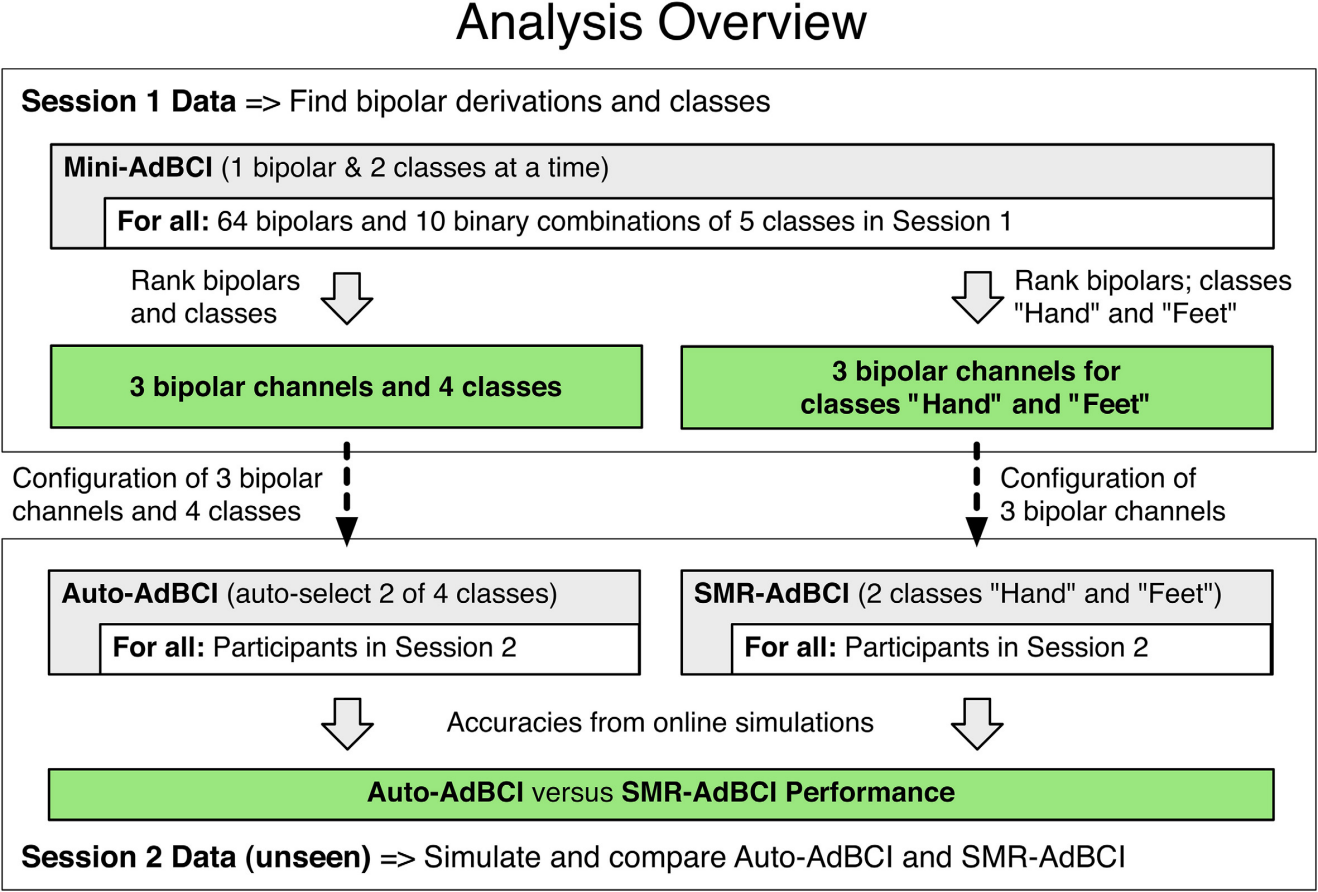
**Procedure to determine whether the *Auto-AdBCI* performs better than a standard *SMR-AdBCI***. The boxes with green background show results.

**Figure 4:**
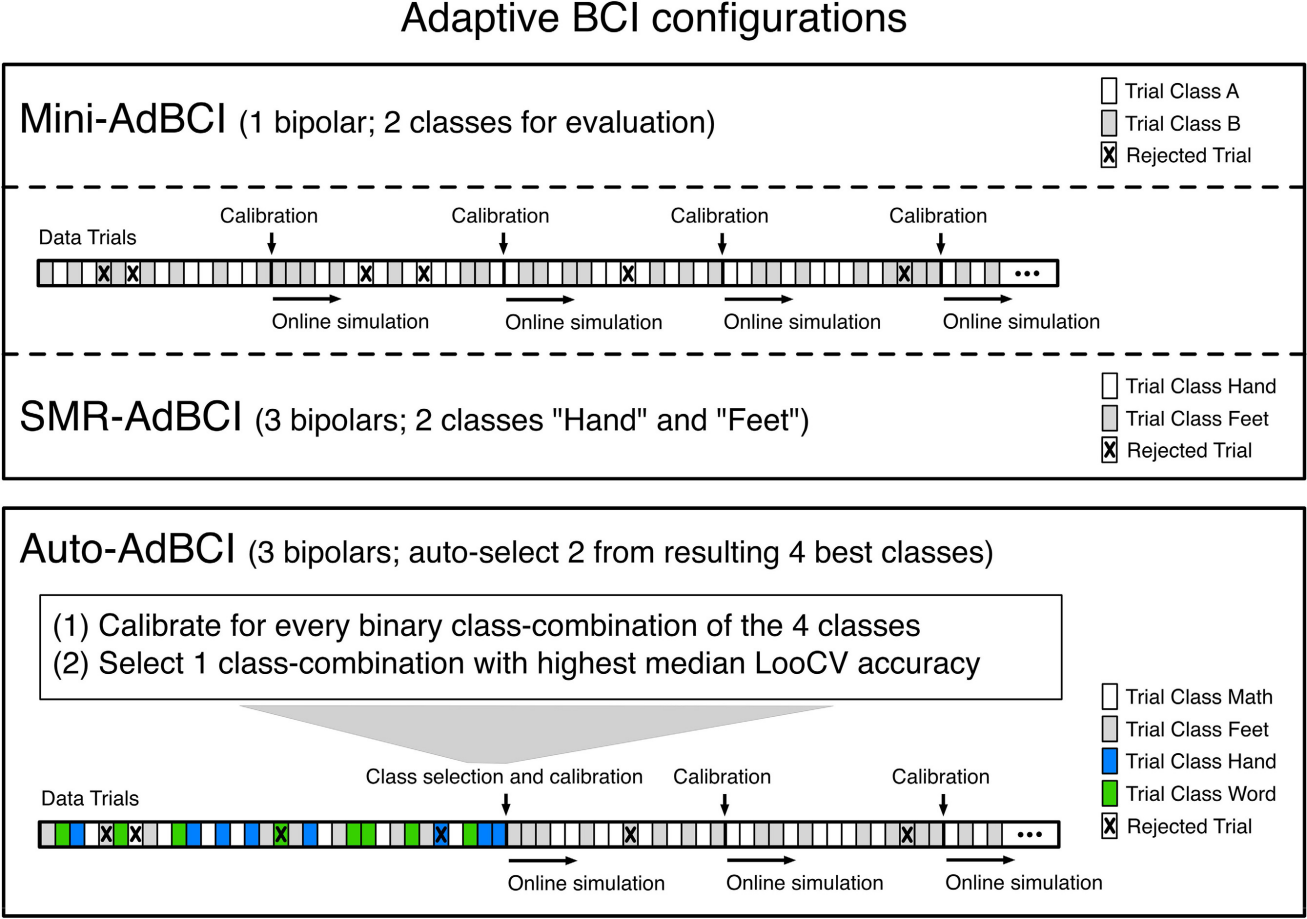
**Overview of the three Adaptive BCI configurations used in our analyses**. The information on which channels and classes were used for each Adaptive BCI configuration is shown in parentheses next to the names of the configurations. The bar in every panel, represents the trials in one session, which the Adaptive BCIs process one by one. The crosses in some trials of the example bars indicate how some trials are removed by the outlier rejection.

To determine three bipolar derivations and four classes for the *Auto-AdBCI* we first simulated the *Mini-AdBCI*—which used only one bipolar derivation and two classes—on all combinations of every single bipolar derivation and every single class combination of all data in Session 1. Figure 3 shows an overview of the analysis and Figure 4 depicts how the different adaptive BCI configurations operate.

In the results of the *Mini-AdBCI* simulation, we ranked the bipolar derivations according to the median (second 4–8 in the trial) of the simulated online accuracy over all class combinations. Inspecting the positions in the resulting list sequentially, starting with the best performing derivation, we then added every bipolar derivation to the result set that did not overlap a scalp area covered by a previously added derivation. From the resulting set of bipolar derivations, we finally selected the top three. For these three bipolar derivations, we selected the four of five classes that on average scored the highest median accuracies.

To determine the three bipolar derivations for the *SMR-AdBCI* we simulated the *Mini-AdBCI* on the classes *Hand* and *Feet* of the data from Session 1 and used the same ranking and selection procedure as for the *Auto-AdBCI*.

To answer our research question, we simulated the previously determined configuration of the *Auto-AdBCI* on the data from Session 2. Likewise, we ran a simulation of the *SMR-AdBCI* configuration, on the same data from Session 2. To avoid over-fitting, we only used the results from the unseen data of Session 2 in our statistical comparison. For the sake of completeness, we also ran both simulations on the seen data of Session 1.

### 2.5 Details on adaptive BCI calibration

Similar to previous implementations (Faller et al., 2012b) the simulated adaptive ERD-based BCIs here (1) collected seven artifact-free trials per class (TPC), (2) did the initial calibration, (3) proceeded to apply the most recent classifier to new trials and (4) re-calibrated on all collected trials, whenever seven new artifact-free TPC were available (see Figure 4). In comparison to Faller et al. (2012b) we reduced the number of initially collected TPC from ten to seven and increased the number of TPC collected between recalibration steps from five to seven. Collecting only seven TPC for initial calibration has proven effective in another previous study (Faller et al., 2012a) and allows our present approach to auto-calibrate in an online setting within 6 min, even though here, we collect data for four instead of two classes. We deem quick auto-calibration very important for usability and practicality, especially in a BCI for end users. From experience with our online Adaptive BCI systems we knew that collecting either five or seven TPC prior to recalibration did not make a difference in efficacy or usability, but here this change was important for practical reasons as it reduced the computational effort for the close to 15000 Adaptive BCI simulations in our analyses.

In this section we explain the classifier “calibration” procedure that is used by all three adaptive BCI configurations and the “class selection and calibration” procedure that is used for initial calibration in the *Auto-AdBCI* (see Figure 4).

For regular classifier calibration, the algorithm first extracted logarithmic band-power features (averaging over 1 s) from every bipolar derivation that was used in this particular adaptive BCI configuration (one or three). Features were extracted for the bands 8–10, 10–13, 13–16, 16–24, and 24–30 Hz. These bands have been previously found to show power modulation in response to performing the specific mental tasks we use (Neuper and Pfurtscheller, 2001; Faller et al., 2012b; Friedrich et al., 2012). From these five features, the system always selected the one with the highest separability in the window from second 4–8 in the trial according to the Fisher criterion (c.f. Bishop, 2007; Faller et al., 2012b).

The system then trained a linear discriminant analysis (LDA, Bishop, 2007) classifier using the selected feature. Here, the system split the time-window from second 4–8 into eight adjacent 0.5 s time-windows and performed leave-one-out cross-validation (LooCV) for every one of them. The window that produced the overall highest median accuracy (second 4–8 in the trial) was used to compute the new classifier, which was from then on used in the simulation.

The *Auto-AdBCI* configuration started collecting data for four instead of two classes. During initial auto-calibration the system then selected two of the four classes in the following way: The *Auto-AdBCI* first performed the regular calibration procedure for every one of the six binary combinations of the four classes and then selected the one class combination, that produced the highest LooCV median accuracy during calibration. If multiple class combinations had the same median LooCV test accuracy, the system picked the class combination whose best feature had a higher separability according to the Fisher criterion.

### 2.6. Outlier rejection

Our adaptive BCI system used trial-based outlier rejection, which worked in multiple phases: First, the method removed outliers by thresholding amplitude and the statistical measures kurtosis and probability of the EEG (Delorme et al., 2007). For the amplitude, the threshold was ± 100 μ*V*. For kurtosis and probability the threshold was ±3.5 times the standard deviation from the respective sample mean. Afterwards, the outlier rejection mechanism iteratively removed trials based on the distribution of the logarithmic band-power for all feature bands (Faller et al., 2012b). This outlier rejection was done separately for the relax period (second 0–3) and the relevant part of the imagery period (second 3–8). The epochs from the relax period were pooled over all conditions, while the imagery period epochs were processed condition specific. The outlier rejection removed on average 12.5 ± 3.1(*SD*)% of the trials. For seven of twelve participants we found some of the lateral channels T3, T4, P7, and P8 to be congested with artifacts. We manually excluded the affected channels for these users prior to analysis.

### 2.7. Performance evaluation and statistics

For system internal model selection and to identify the most effective bipolar derivations and classes in our analyses, we rely on the median accuracy between second 4 and 8 in the trial of the simulated online accuracy as a performance measure. For these purposes, this measure has proven robust and reliable (Faller et al., 2012b). To measure final simulated online BCI performance, however, high accuracy in a much shorter time window is relevant. Krausz et al. (2003) for example, showed how in a “Basket Paradigm,” the trial length can be optimized for each user to increase BCI performance. For the final results, we therefore report the peak accuracy within the window from second 4–8 in the trial. Assuming a conservatively low number of 30 TPC in the online simulation, the level of better than chance accuracy for a significance level of *p* = 0.01 in a binary decision task is 66.7% (Müller-Putz et al., 2008). To test the difference hypothesis of our research question we conducted a mixed design repeated measures analysis of variance (ANOVA) with one between-subject factor “Pathology” (2 levels, SCI and Stroke), one within-subject factor “BCI-Type” (2 levels, *Auto-AdBCI* and *SMR-AdBCI*) and the dependent variable “Simulated online peak accuracy.” We examined the two main effects and their interaction on the results from Session 2. Normal distribution was confirmed by the Kolmogorov-Smirnoff test and Greenhouse-Geisser Epsilon was used for correction. We considered *p*-values smaller than 0.05 statistically significant.

## 3. Results

### 3.1. Channels and classes for the *SMR-AdBCI* and the *Auto-AdBCI*

**Figure 5:**
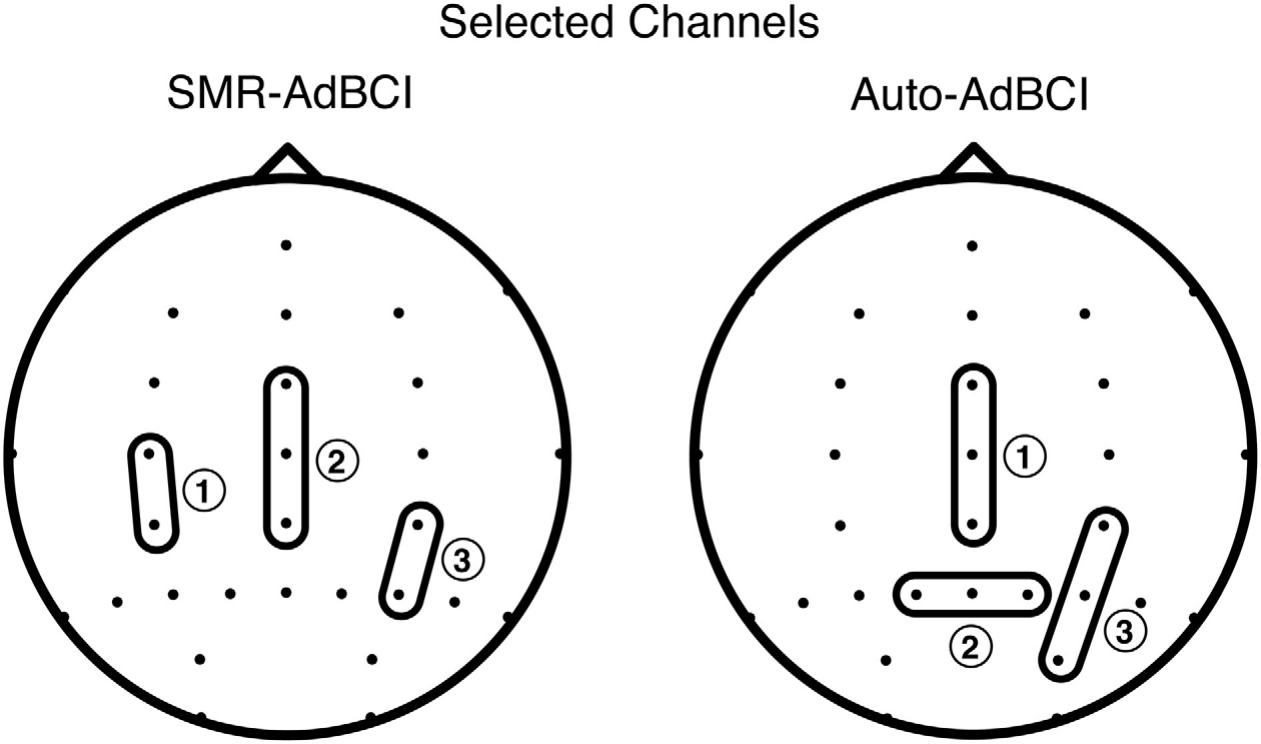
**The selected bipolar derivations for the *SMR-AdBCI* and the *Auto-AdBCI* system**. The annotated numbers show the ranking of the bipolars, with number one performing the best.

In the analyses on the data of Session 1 we identified the bipolar derivations at Cz (FCz-CPz), Pz (P1-P2), and P4 (CP4-PO4) (see Figure 5) to produce the highest accuracy. Over these three selected channels we further found the mental tasks *Math, Feet, Hand*, and *Word* to perform best, leading us to reject class *Nav*. When limiting the classes to *Hand*, and *Feet* for the *SMR-AdBCI* we identified the bipolar derivations C3-CP3, again Cz (FCz-CPz), and CP4-P4 (see Figure 5) to produce the highest accuracy.

### 3.2. Performance of the *Auto-AdBCI*

By BCI-Type, we found an overall peak accuracy of 75.7 ± 8.4 (*SD*)% for the *Auto-AdBCI* system and an overall peak accuracy of 66.3 ± 7.2 (*SD*)% for the *SMR-AdBCI* system. That means the performance of the *Auto-AdBCI* was 9.4% accuracy higher than that of the *SMR-AdBCI*. This difference was statistically significant [*F*_(1, 7)_ = 15.705, *p* < 0.01]. The *Auto-AdBCI* system worked significantly better than chance for eight of nine users, while the *SMR-AdBCI* system worked significantly better than chance for six of nine users (*p* < 0.01, Müller-Putz et al., 2008).

By Pathology, we found an overall peak accuracy of 75.6 ± 7.0 (*SD*)% for users with SCI and 65.2 ± 8.1 (*SD*)% for users with stroke. That means the average performance of both BCI-Types is 10.4% higher for users with SCI than for those with stroke. This difference was statistically significant [*F*_(1, 7)_ = 10.406, *p* < 0.05]. There was no statistically significant effect of the interaction of Pathology and BCI-Type on the peak accuracy [*F*_(1, 7)_ = 0.017, ns].

**Figure 6:**
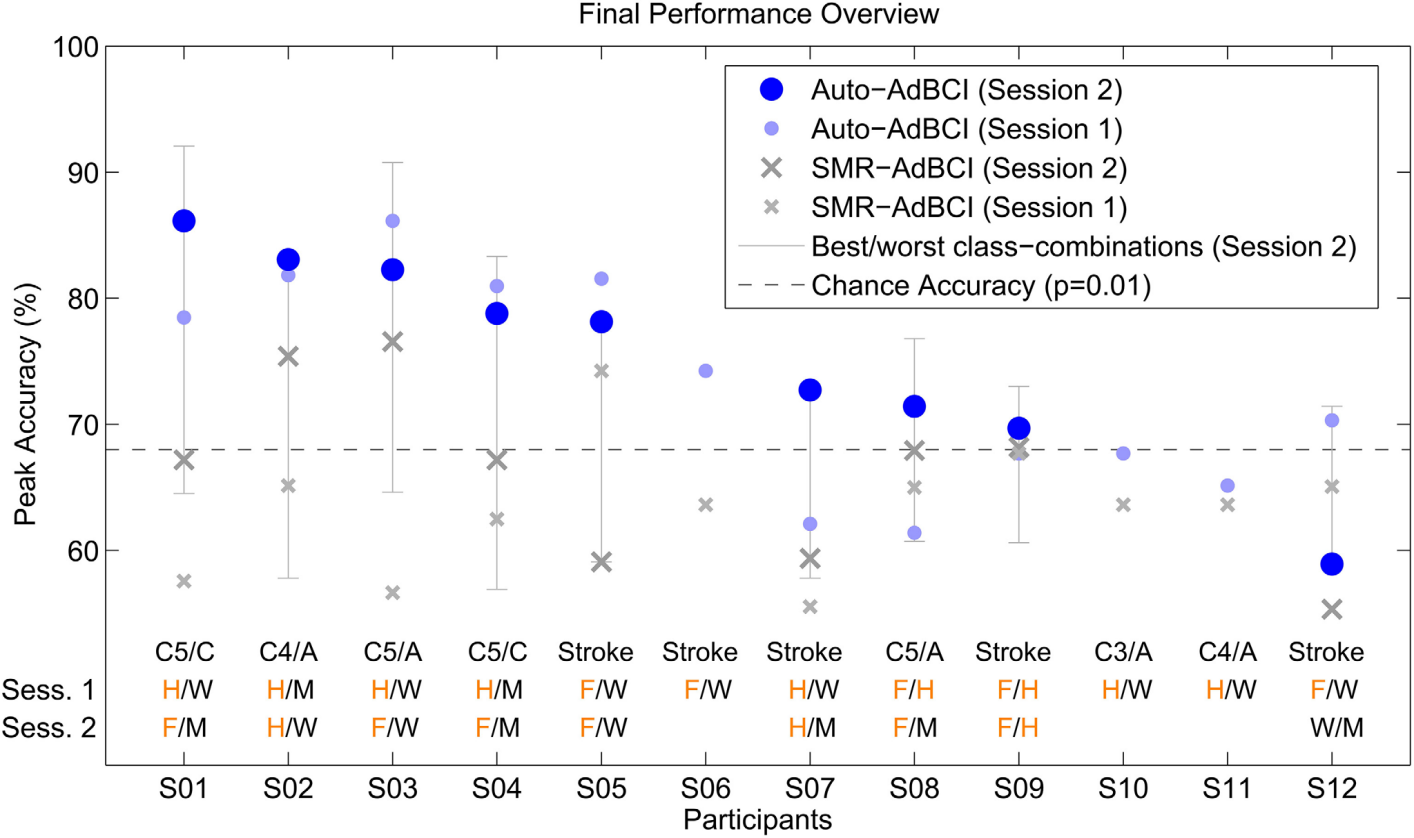
**Performance overview for the *Auto-AdBCI* and the *SMR-AdBCI* configuration**. The light and dark blue dots show the simulated peak accuracies for the *Auto-AdBCI* on the seen data from Session 1 and the unseen data from Session 2. The light gray whiskers indicate the span between best and worst possible class-combinations for the unseen data of Session 2. The small and large gray crosses show the simulated peak accuracies of the *SMR-AdBCI* on the seen data of Session 1 and the unseen data of Session 2 respectively. The first of the three lines at the bottom indicates pathology. The second and third show the class-combinations auto-selected by *Auto-AdBCI* in Session 1 and Session 2. The single letters are abbreviations for the classes *Feet* (F), *Hand* (H), *Word* (W), and *Math* (M). Letters in orange indicate motor-related mental tasks, while letters in black indicate non motor-related mental tasks.

Figure 6 shows the peak accuracies for the simulations of the *Auto-AdBCI* and *SMR-AdBCI* systems on the seen data of Session 1 and the unseen data of Session 2. Table 2 shows the simulated online peak accuracies, separately for the two sessions and pathologies.

**Table 2:** **Simulated online peak accuracies for sessions and pathologies**.

		**Peak accuracies for different Adaptive BCI configurations**			
		**Best class-combination**	**Auto-AdBCI**	**SMR-AdBCI**	**Worst class-combination**
Session 1^†^	Stroke	73.2	71.2	65.2	57.6
	SCI	80.8	74.5	62.0	60.2
	Mean (*SD*)	77.6 (6.1)	73.1 (8.5)	63.4 (5.1)	59.1 (2.5)
Session 2	Stroke	73.9	69.9	60.5	59.1
	SCI	85.3	80.3	70.8	60.9
	Mean (*SD*)	80.2 (7.7)	75.7 (8.4)	66.3 (7.2)	60.1 (2.8)
Mean (*SD*)		78.4 (6.1)	73.6 (7.7)	64.5 (3.5)	59.5 (2.1)

The *Auto-AdBCI*, initially auto-selected one of six class combinations according to a heuristic. Based on seven trials per class, the heuristic tried to select a class-combination that would allow for a highest possible peak control accuracy over the session. To compare, we simulated the overall session accuracy not only with the auto-selected class-combination (*Auto-AdBCI*), but also with all other class-combinations. The column “Best Class-Combination” is the average when considering for every user only the one class-combination that eventually produces the highest overall accuracy. In analogy, the column “Worst Class-Combination” considers for every user only the one class-combination that eventually produces the lowest overall accuracy.

† *Notice, the data of Session 1 is “seen data” as it has been previously used to determine the configurations of the BCIs*.

## 4. Discussion

Our findings support our hypothesis: In our sample of nine individuals with SCI or stroke in Session 2, auto-selecting a user specific class combination of motor-related and non motor-related mental tasks during initial calibration of an adaptive ERD-based BCI significantly increased performance in comparison to an adaptive ERD-based BCI that used only motor-related mental tasks.

### 4.1. Performance of the *Auto-AdBCI*

The *Auto-AdBCI* successfully auto-calibrated and adapted to the patterns of oscillatory brain activity of the users with severe motor impairment in our study. On the unseen data of Session 2, a high number of eight of nine users performed better than chance. For seven of nine users the system performed higher than 70% accuracy which had previously been found necessary to effectively operate a spelling application (Kübler et al., 2001).

Figure 6 shows how the simulated performance of the *Auto-AdBCI* configuration on the unseen data of Session 2 (dark blue dots) is in most cases very close to that of the best possible class combination (upper end of gray whiskers), which indicates that our comparably simple auto-selection heuristic was overall very effective. The simulated online accuracy of the *Auto-AdBCI* on the unseen data of Session 2 was less than 5% lower than an average with the best-possible class-combinations but more than 15% better than an average with the worst-possible class-combinations (see Table 2). In over 80% of all sessions, the *Auto-AdBCI* selected a class-combination where one class was either *Hand* or *Feet* and the other class was either *Word* or *Math*. The less than 20% of all sessions where the *Auto-AdBCI* selected class-combinations where both tasks were either only motor-related or non motor-related are with the five of twelve users for whom the system worked least effectively. From the gray whiskers in Figure 6 we see, that, at least in Session 2, none of the other class-combinations perform substantially better, which indicates that this is not a problem with the heuristic approach of the *Auto-AdBCI*. With respect to the selected class-combinations, we found no indication that there may be a systematic difference between the pathologies SCI and stroke.

Our analyses again highlighted some important points to keep in mind for bringing BCIs to end-users. For example the issue with artifactual activity in the EEG of users with motor impairment: After we had to remove one or more artifact congested lateral EEG channels in the data of more than half of the participants, the automatic outlier rejection of our system still had to remove on average 12.5% of the trials. The other issue is that users with severe motor impairment often are also more susceptible to illness, have limited mobility and independence and are therefore more likely to miss BCI training sessions.

### 4.2. Comparing to other studies that involved users with SCI or stroke

High inter-subject variability in EEG studies and differences in the used paradigms make a detailed comparison to independent population samples in other BCI studies difficult. In addition, most previous BCI studies involving individuals with SCI or stroke did not consider non motor-related mental tasks but instead focused mostly on motor-related tasks. We therefore decided to check whether the performance of the *SMR-AdBCI* which we used as baseline, is comparable to the results of existing studies. If the performance of the *SMR-AdBCI* is comparable to other systems, then this supports the findings in our study, that the *Auto-AdBCI* does perform better than a purely motor imagery based system. We compare results of other studies to the result of the *SMR-AdBCI* on the unseen data of Session 2. We consider higher performance better, but a high number of sensors less practical for home or clinical use with impaired end users.

For end users with SCI, Pfurtscheller et al. (2000) and Müller-Putz et al. (2005) showed effective ERD-based BCI control based on motor-related tasks in early case studies. Later, Pfurtscheller et al. (2009) found an overall accuracy result, lower than that of our *SMR-AdBCI* (61.7 vs. 70.8%) in offline analyses on seven individuals with SCI using 16 electrodes instead of 6 in our setup. Conradi et al. (2009) found a higher overall accuracy of 75% in four tetraplegic volunteers, but they used 64 instead of 6 electrodes and screened the participants from a larger group, which makes the results incomparable. In a recent study, Rohm et al. (2013) found an accuracy result comparable to our *SMR-AdBCI* (65.7 vs. 70.8%) in ten individuals with SCI over a large number of sessions.

For end users with stroke, Mohapp et al. (2006) found accuracy results in ten hemiparetic individuals, that were comparable with those of the *SMR-AdBCI* (67.1 vs. 60.5%). The minor differences could be explained by the stronger impairment of the participants in our sample. In a study involving eight stroke survivors, Buch et al. (2008) found an overall accuracy of 52.8% (median) in the first session and an overall end-accuracy of 72.5% (median) after 20 sessions of training. We deem the overall accuracy of 59.2% (median) we found with the *SMR-AdBCI* in Session 2 comparable. More recently, Ang et al. (2011) found a higher overall accuracy of 74% in a large sample of 54 stroke survivors, but they used a higher number of electrodes (27 instead of 6).

We find that the *SMR-AdBCI* performs at a similar level as comparable ERD-based BCI systems with users with similar pathology. This supports our main finding, that the *Auto-AdBCI* performs better than a standard adaptive BCI that relies only on motor tasks.

### 4.3. Analysis of class separability patterns

Overall, but especially in users with SCI, we found higher class separability as soon as non motor-related mental tasks were involved, which explains the overall significantly higher performance in the *Auto-AdBCI* when compared to the *SMR-AdBCI* (75.7 vs. 66.3% peak accuracy). In addition, we found stronger class separability in the group SCI as compared to the group Stroke, which is also reflected in the results of our statistical performance comparison. It is interesting to note, that the patterns of separability in the group SCI show distinct spatio-spectral differences to those of the healthy controls. The patterns in the group Stroke, are more similar to those of the healthy controls.

**Figure 7:**
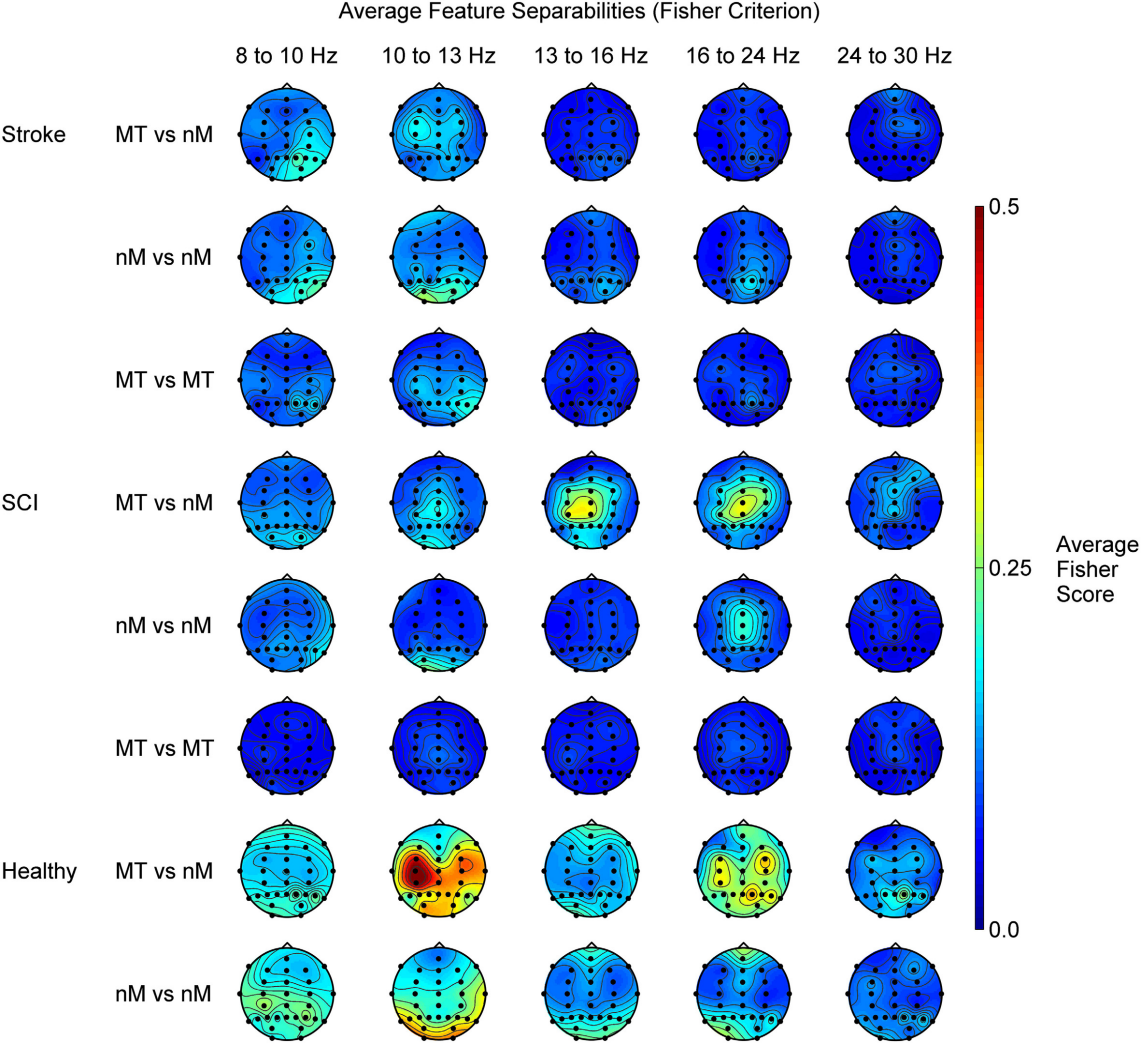
**Topographic projections of the average feature separabilities (Fisher criterion) for the dimensions pathology, class combination type and frequency band**. The abbreviations “*MT*” and “*nM*” stand for motor-related and non motor-related mental tasks respectively. The row *MT* vs. *nM* for example shows an average over all class combinations where one class is a motor-related and the other one is a non motor-related mental task. The data for group Healthy, did not include the class *Feet*. For the class combinations *MT* vs. *nM* and *nM* vs. *nM*, for the users with motor impairment we therefore excluded the class *Feet*. The data is averaged across 2 sessions for 9 healthy users (Friedrich et al., 2012) and 12 with severe motor impairment.

Figure 7 shows topographical projections of feature separabilities (Fisher criterion) after outlier rejection for different class combinations, frequency bands and user groups (SCI, Stroke and Healthy). The data set of the healthy individuals is from a similar study (Friedrich et al., 2012). That study included all the mental tasks used here, except the second motor task *Feet*.

In the group SCI, we found interesting differences to the groups Stroke and Healthy: Most importantly, for the combination of motor-related and non-motor related mental tasks we found strong, topographically focal separability around the vertex, most prominent in the feature bands 13–16 Hz and 16–24 Hz. For task combinations of non-motor related mental tasks we found a similar, spatio-spectrally even more focal pattern of separability between 16 and 24 Hz.

These observations are in accordance with reports in literature: Curt et al. (2002), Alkadhi et al. (2005), Conradi et al. (2009) and most recently Gourab and Schmit (2010) found performing motor tasks to cause increased but more diffuse activity in cortical motor areas (including increased central beta ERD) in individuals with SCI when compared to healthy controls. Curt et al. (2002) suggested that this phenomenon might be a result of “sprouting or rewiring” which “may occur close to the SCI segments.” This would also explain the differences in the separability patterns when comparing to the groups Healthy and Stroke. In the groups Healthy and Stroke, the spinal cord is in tact and such “sprouting” would therefore not occur. Gourab and Schmit (2010) on the other hand speculated, that the increased ERD activity they found in users with SCI during attempted execution of a foot movement would be due to “increased difficulty in attempting movement with the paralyzed extremity.” For the purpose of BCI operation, it is relevant to note, that the patterns of SCI survivors seem to show stronger class separability when involving non motor-related mental tasks than when only motor-related mental tasks are used.

In the group Stroke we found activation patterns that are weaker but otherwise similar to those in the group Healthy. This is in accordance with earlier studies, which found motor-related tasks in individuals with stroke to produce similar patterns of separability as in healthy controls (Mohapp et al., 2006; Ang et al., 2008; Buch et al., 2008; Sharma et al., 2009). Our present study confirms the similarity of the separability patterns between healthy users and individuals with stroke now also for task combinations that involve non motor-related mental tasks.

### 4.4. Limitations and future prospects

A limitation of the present study is that the results were obtained through offline analyses. Tests with online implementations will show whether non-motor related mental tasks like “Word” or “Math” are also practical for real world applications. Another limitation of the present system is performance: Our system showed significantly improved accuracy over previous approaches. Still, an average of 70–75% accuracy may not be enough to attain satisfactory control in a real world setting for many end users. Based on previous findings involving online ERD-based adaptive BCIs (Vidaurre et al., 2006, 2011; Faller et al., 2012b) we are hoping to see the additional closed-loop feedback lead to even higher system performance, especially with training over multiple sessions. As a next step it will be important to explore whether the advantages of the presented approach also translate to user populations with severe motor impairment as a result of medical conditions other than SCI or stroke, like amyotrophic lateral sclerosis (Kübler and Neumann, 2005) or cerebral palsy (Neuper et al., 2003). In another research direction, it would be interesting to evaluate, whether adaptive ERD-based BCIs could be useful tools for neuro-rehabilitation (Dobkin, 2004; Daly and Wolpaw, 2008) after neural injuries like stroke (Grosse-Wentrup et al., 2011), SCI (Cramer et al., 2007) or other neurological disorders.

## Conclusion

In our sample of nine individuals with SCI or stroke, auto-selecting a user specific class combination of motor-related and non motor-related mental tasks during initial calibration of an adaptive ERD-based BCI significantly increased performance in comparison to an adaptive ERD-based BCI that used only motor-related mental tasks. This could have very strong implications on the use of ERD-based BCIs, especially for clinical applications: As of now, most BCI protocols still exclusively rely on motor-related mental tasks. Our findings show that including non motor-related mental tasks can significantly improve performance for potential end users with SCI or stroke.

## Author contributions

Josef Faller, Reinhold Scherer, and Elisabeth V. C. Friedrich contributed equally in conceiving the experiment. Reinhold Scherer implemented the data acquisition system. Josef Faller performed all data analyses. Josef Faller, Elisabeth V. C. Friedrich, Ursula Costa, Eloy Opisso, and Josep Medina collected the data. Josef Faller, Reinhold Scherer, Eloy Opisso, Josep Medina, and Gernot R. Müller-Putz wrote the manuscript.

## Funding

This work was supported by the FP7 EU Projects BrainAble (No. 247447) and BackHome (No. 288566). This paper only reflects the authors' views and funding agencies are not liable for any use that may be made of the information contained herein.

### Conflict of interest statement

The authors declare that the research was conducted in the absence of any commercial or financial relationships that could be construed as a potential conflict of interest.
